# Supplementary material for: Development and Validation of the Adolescent Sexual and Reproductive Competency Assessment Tool (ASRH-CAT) for Healthcare Providers
Source: Healthcare (Basel). 2023 Apr 13;11(8):1116. doi: 10.3390/healthcare11081116 (PMC10138378; doi:10.3390/healthcare11081116)
Supplement: Supplementary file 1 [file healthcare-11-01116-s001.zip › healthcare-2175181-supplementary.pdf]

Table S1. MANAGEMENT AND DECISION-MAKING IN ADOLESCENT SEXUAL AND REPRODUCTIVE HEALTH COMPETENCY ASSESSMENT TOOL (ASRH_CAT)

INSTRUCTION:

This survey asks for your knowledge, opinion, and your intention in providing services management on adolescents' health related to sexual and reproductive health concerns. It helps you to suggest the level of competency you need to manage and coordinate for ASRH at the primary health care clinics and community. Answer each question by ticking (√) the most appropriate to your understanding (only one box per question). If you are uncertain about how to answer a question, please give the best answer you can.

Scale: 0-4 (0-strongly not confident; 4-strongly confident)

**Domain 1: Self-perceived ability in providing ASRH education** (think about your own perception of the ability to provide health education to adolescent clients)			Strongly not	Not confident	Not sure	With confident	strongly confident
I can provide health education to the adolescent client regarding the scope as listed:							
1	HE1	ASRH service access.					
2	HE2	Availability of Sexually Transmitted Diseases (STI) screening test.					
3	HE3	HIV screening test service.					
4	HE4	Information on abortion					
5	HE5	Contraception methods, including emergency contraceptive measures for adolescents.					
6	HE6	Guide on proper condom usage.					
7	HE7	STI prevention measures.					
8	HE8	HIV prevention measures.					
9	HE9	Reproductive system development in adolescents.					
10	HE10	Adolescent services including procedures and management at the clinic					
11	HE11	Information on risk sexual activities prevention and consequences					
12	HE12	Gender identity and sexual orientation (including lesbian, gay, bisexual, and transgender)					
13	HE13	safe sex practice					
**Domain 2: Self-perceived capability in ASRH management** (Self-perceived capability in ASRH managing and decision-making skills according to guideline practice standards for PHC)							
I am capable of to…							
14	C1	take SRH history from an adolescent by exploring possible undisclosed issues.					
15	C2	explain the procedure involved in adolescent health services to the client					
16	C3	conduct a physical examination when needed for adolescent growth and development assessment					
17	C4	provide a trustful consultation with rights to privacy and confidentiality					
18	C5	seek consultation before any referral made					
19	C6	adhere to local policy and guidelines of ASRH.					
20	C7	provide pregnancy care for adolescents if needed.					
21	C8	provide contraceptive services, including emergency contraceptive measures to adolescents if needed.					

22	C9	provide a treatment plan for STIs in adolescents if needed.					
23	C10	I can communicate with all the stakeholders about the value of providing respectful, confidential health services to adolescents.					
24	C11	provide appropriate care for a sexually abused adolescent.					
**Domain 3: Self-perceived adequate ASRH knowledge in decision making** (self-perceived knowledge required for ASRH services/managing program)							
I know how to…….							
25	K1	determine appropriate diagnostic tests, including for sexual assault cases.					
26	K2	diagnose pregnancy in adolescents, including interpreting pregnancy tests.					
27	K3	diagnose STI in adolescent					
28	K4	perform appropriate timing to do clinical procedures					
29	K5	prepare the adolescent client for examination procedures					
30	K6	use the standards protocols underpinning care in ASRH.					
31	K7	advice usage of various contraceptive methods					
32	K8	address related factors that influence ASRH care delivery decision-making (e.g. age, gender, policies).					
33	K9	Apply the ASRH-related laws i.e. in Malaysia the Child Act (2001) and Child Act Amendment (2016)					
34	K10	Apply the local policies for the provision of ASRH service.					
35	K11	Assess for ASRH risk factors					
**Domain 4: Self-perceived appropriate attitude ASRH management** (self-perceived attitudes toward ASRH clients)							
I am …							
36	A1	not judge adolescents with societal SRH problems such as pregnancy out of wedlock, HIV positive status, having STI, practicing different sexual orientations, or are gender dysphoria					
37	A2	ready to provide appropriate treatment services to all adolescent clients with SRH problems regardless of their background					
38	A3	able to communicate SRH issues with adolescents.					
39	A4	able to give non-judgemental counseling to an adolescent who has been sexually abused.					
40	A5	not take the adolescent's SRH issues personally.					

ASRH_CAT has 40 items and 4 domains with a range of scores 0-100. Each domain is calculated independently. A higher score indicates a better competency level. The calculation for each domain is as follows:

Domain 1(HE1-HE13): Self-perceived ability in providing ASRH education: sum scores item in domain 1 X 100/(13x4)
Domain 2(C1-C11): Self-perceived capability in ASRH management: sum scores item in domain 2 X 100/(11x4)
Domain 3(K1-K11): Self-perceived adequate ASRH knowledge in decision making: sum scores item in domain 3 X 100/(11x4)
Domain 4 (A1-A5): Self-perceived appropriate attitude in ASRH management: sum scores item in domain 4 X 100/(5x4)

Aggregation scores for all domains are not calculated as the competency level needs to be assessed based on domains.
